# Deafness Gene Expression Patterns in the Mouse Cochlea Found by Microarray Analysis

**DOI:** 10.1371/journal.pone.0092547

**Published:** 2014-03-27

**Authors:** Hidekane Yoshimura, Yutaka Takumi, Shin-ya Nishio, Nobuyoshi Suzuki, Yoh-ichiro Iwasa, Shin-ichi Usami

**Affiliations:** Department of Otorhinolaryngology, Shinshu University School of Medicine, Matsumoto, Nagano, Japan; University of Salamanca- Institute for Neuroscience of Castille and Leon and Medical School, Spain

## Abstract

**Background:**

Tonotopy is one of the most fundamental principles of auditory function. While gradients in various morphological and physiological characteristics of the cochlea have been reported, little information is available on gradient patterns of gene expression. In addition, the audiograms in autosomal dominant non syndromic hearing loss can be distinctive, however, the mechanism that accounts for that has not been clarified. We thought that it is possible that tonotopic gradients of gene expression within the cochlea account for the distinct audiograms.

**Methodology/Principal Findings:**

We compared expression profiles of genes in the cochlea between the apical, middle, and basal turns of the mouse cochlea by microarray technology and quantitative RT-PCR. Of 24,547 genes, 783 annotated genes expressed more than 2-fold. The most remarkable finding was a gradient of gene expression changes in four genes (*Pou4f3*, *Slc17a8*, *Tmc1*, and *Crym*) whose mutations cause autosomal dominant deafness. Expression of these genes was greater in the apex than in the base. Interestingly, expression of the *Emilin-2* and *Tectb* genes, which may have crucial roles in the cochlea, was also greater in the apex than in the base.

**Conclusions/Significance:**

This study provides baseline data of gradient gene expression in the cochlea. Especially for genes whose mutations cause autosomal dominant non syndromic hearing loss (*Pou4f3*, *Slc17a8*, *Tmc1*, and *Crym*) as well as genes important for cochlear function (*Emilin-2* and *Tectb*), gradual expression changes may help to explain the various pathological conditions.

## Introduction

The auditory systems of mammalians that perceive sounds are organized based on the separation of complex sounds into their component frequencies (tonotopy). Tonotopy begins at the level of the auditory sensory epithelium where specific frequencies are distributed along the tonotopic axis of the mammalian cochlea [1].

Gradients in morphological and physiological characteristics of the inner ear in different species have also been reported [1]. In addition, gene expression gradients along the tonotopic axis in chicken auditory epithelium have been also reported [2]. However, few reports are available on mammalian gene expression gradients.

Hearing loss that disturbs normal communication is a common sensory disorder worldwide. Most congenital or childhood onset hearing impairments are non syndromic. As of Apr. 2013, 27 dominant, 40 recessive and 3 X-linked genes whose mutations cause non syndromic hearing loss have been reported according to the Hereditary Hearing loss Homepage (http://hereditaryhearingloss.org/).

Interestingly, the audiograms of autosomal dominant non syndromic hearing loss (ADNSHL) can be distinctive, and thus useful to identify the gene responsible [3]. For example, mutations in *WFS1* are found in 75% of families with dominantly inherited hearing loss that initially affects the low frequencies while sparing the high frequencies [4], [5]. On the contrary, many of the mutations in ADNSHL, like *KCNQ4*, *DFNA5*, *POU4F3*, and *SLC17A8*, affect the high frequencies [6]. However, the mechanism that accounts for the distinct frequency patterns has not been clarified. We hypothesized that certain gene expression patterns might show a gradient within the cochlea that could, at least in part, correspond with the distinct shapes of audiograms in ADNSHL.

Microarray analysis, which provides whole gene expression data, can be used to analyze differential gene expression among tissues [7]. In this study, to analyze the mechanism of the distinct audiograms in ADNSHL, we examined and compared gradient gene expression profiles, in particular ADNSHL genes, between the apical, middle, and basal turns of the cochlea by microarray technology.

## Materials and Methods

### Tissue dissection and RNA extraction

**Figure 1:**
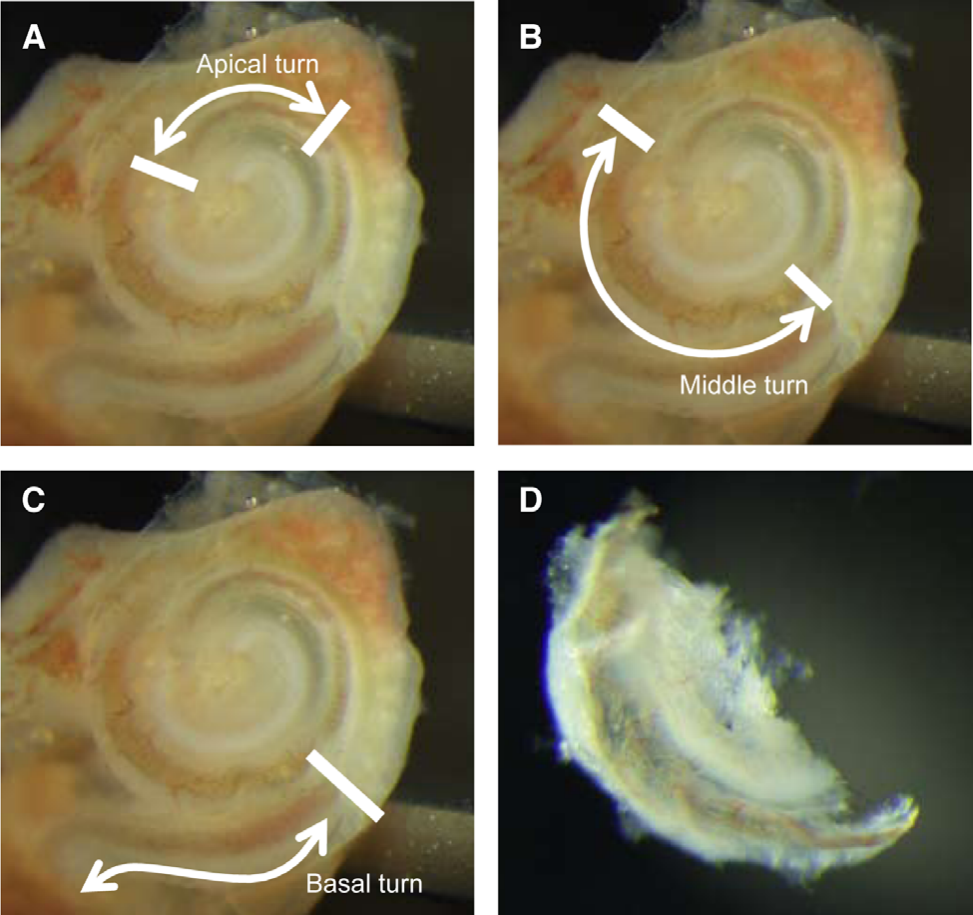
Microscopical image of the mouse cochlea (right ear). Bars indicate the incision points for each turn sample. A: apical turn, B: middle turn, C: basal turn, D: dissection example

Four C57BL/6 mice aged 6 weeks were euthanized by decapitation under deep anesthesia induced by an intraperitoneal injection containing 75 mg/kg Ketamine (Daiichi Sankyo, Tokyo, Japan) and 32.4 mg/kg Pentobarbital Sodium (Kyoritsu, Tokyo, Japan). Inner ears were rapidly extracted from the temporal bone and transferred into RNA*later* solution (Ambion, Austin, TX, USA). After removing the otic capsule, the cochlea including the lateral wall comprising the stria vascularis, spiral ligament, and spiral prominence, the organ of Corti and the spiral ganglion neurons were dissected and separated into the apical, middle and basal turns (Fig. 1). All of these dissections were performed in RNA*later* solution to prevent RNA degradation. Total RNA was were extracted using the QIAGEN RNeasy Mini Kit (QIAGEN, Hilden, Germany) according to the manufacturer's protocol. The quality of the extracted total RNA was assessed with the Agilent 2100 Bioanalyzer (Agilent Technologies, Waldbronn, Germany) and found to be adequate for microarray analysis (data not shown).

### RNA labeling and purification

Total RNA (25 ng each) was reverse transcribed with the Low Input Quick Amp Whole Transcriptome Labeling Kit (Agilent Technologies). After reverse transcription process, labeled cRNA was synthesized from cDNA by using T7 RNA polymerase mix and cyanine 3-CTP according to the manufacturer's instructions. Labeled cRNA was purified using the Rneasy Mini kit (QIAGEN).

### Microarray hybridization

To analyze gene expression of each cochlea turn, 12 SurePrint G3 Mouse Exon Microarrays (Agilent Technologies), which were spotted with 165,984 exon probes (24,547 genes), were hybridized to labeled cRNA (4 microarrays were used for each turn sample). Prior to the hybridization step, Cyanine 3-labeled cRNAs were fragmented using 25X fragmentation buffer at 60°C in a water bath for 30 min and then hybridized to a microarray slide for 17 hours at 65°C in a hybridization oven and washed using Gene Expression Wash Buffer (Agilent Technologies).

### Microarray scanning and statistical analysis

Fluorescence intensities were measured with the Agilent Microarray Scanner (Agilent) using the scanning protocols specific for each microarray assay and raw microarray image files were created. The expression data were extracted from raw microarray image files using Agilent Feature Extraction Image Analysis Software (Version 10.7.3.1). The software also generated quality control reports using the protocol specific for the microarray assays as well as data files for analysis with GeneSpring GX (Version 11, Agilent Technologies). Signal intensities for each probe were normalized to the 75th percentile without baseline transformation.

Data for each microarray was analyzed using the manufacturer's workflow in GeneSpring GX. For gene-level analysis, the average expression levels of each exon probe were used. Then averages of four microarray data of each cochlea turn (base, middle, and apex) were used for comparison analysis (one-way analysis of variance (ANOVA)) by using GeneSpring GX. The microarray data have been lodged in the Gene Expression Omnibus (http://www.ncbi.nlm.nih.gov/geo/) as accession number: GSE53863.

### Quantitative RT-PCR

To confirm the microarray analysis results, qPCR was performed on 9 deafness genes. Reverse transcription was performed with 4 total RNA samples of each cochlea turn by using High Capacity RNA-to-cDNA Kit (Life Technologies, Foster City, CA, USA) as described in the manufacturer's procedure. The TaqMan probe for each gene was selected from the TaqMan Gene Expression Assay system (https://products.appliedbiosystems.com/ab/en/US/adirect/ab?;cmd=ABGEKeywordSearch,Life Technologies). *Gapdh*, *Actb*, *Rps17*, *Rpl30*, *Atp6*, and *Ipo8* were chosen as internal control genes. The estimated gene expression level (EL) was normalized to the internal control gene expression level and data are presented as the mean of log_2_EL.

### Ethics Statement

All experimental procedures were performed in accordance with the regulations for animal experimentation of Shinshu University. These experiments were approved by Shinshu University institutional animal care and use committee.

## Results

### Scatter plot analysis

**Figure 2:**
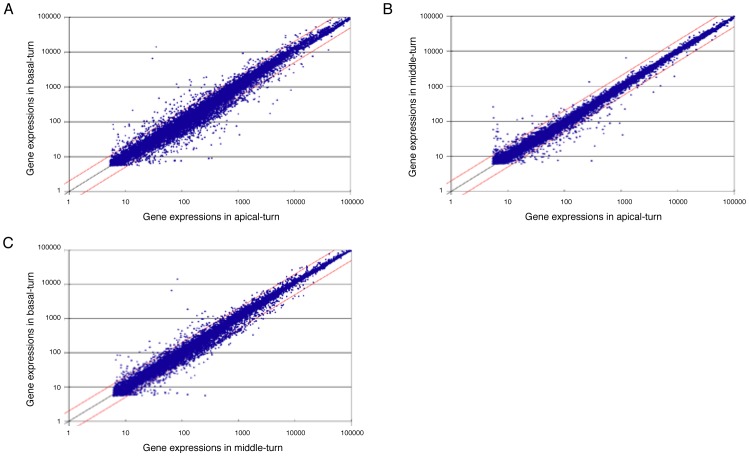
Scatter plot analysis of gene expression profile of each cochlear turn. Black lines indicate equal gene expression and red lines indicate 2-fold gene expression. A: apical turn vs. basal turn, B: apical turn vs. middle turn, C: middle turn vs. basal turn.

To confirm the technical stability of cochlear dissection and RNA extraction and to estimate global gene expression change, we performed scatter plot analysis of the gene expression profiles of each cochlear turn. In each comparison of basal, middle and apical cochlear turns, the gene expression patterns were quite similar and most gene expression changes were less than 2-fold (Fig. 2).

In detail, the gene expression profile of the apical turn of the cochlea was more similar to that of the middle turn than the basal turn. The scatter plot of the apical turn vs. basal turn showed lower correlation than the others. From these results, the gene expression of each cochlear turn clearly indicated gradual gene expression change according to the tonotopic axis.

### Genes indicate differential expression in each cochlear turn

To analyze difference in gene expression, we focused on the genes, which were expressed 2-fold or more in one turn than in the other turn. Each of the gene expression levels was estimated from the average value of four microarray results for independent mouse samples and one-way ANOVA was employed before the comparative analysis as written in the material and methods. Of 24,547 genes, 941 differed more than 2-fold. Of these genes, 783 genes (3.2%: 783/24547) had been annotated and the others were predicted genes (Table S1).

Out of the 783 annotated genes, 747 were differentially expressed between apex and base; 51 were differentially expressed between apex and middle, and 458 genes were differentially expressed between middle and base (some genes were in more than one group) (Table 1). This is consistent with the notion that those genes whose expression changes showed an apical-to-basal gradient along the tonotopic axis. The complete list of differentially expressed genes for each of the three comparisons (i.e., apex vs. base, apex vs. middle, middle vs. base) is indicated as a supporting information file (Tables S2, S3, and S4).

**Table 1:** The numbers of differentially expressed genes for apex vs. base, apex vs. middle, middle vs. base.

apex vs. base		apex vs. middle		middle vs. base	
up in apex	up in base	up in apex	up in middle	up in middle	up in base
571/783	176/783	38/783	13/783	389/783	69/783
747/783		51/783		458/783	

### Overall tonotopic expression pattern

Most (96.2%) genes expression changes did not differ and only a limited number of genes showed tonotopic expression pattern.

### Tonotopic expression of the genes responsible for hearing loss

The results of gene expression analysis of each cochlear turn showed that 4 ADNSHL genes (*Pou4f3*, *Slc17a8*, *Tmc1*, and *Crym*) and 9 autosomal recessive non syndromic hearing loss genes (*Otof*, *Strc*, *Ush1c, Pcdh15*, *Grxcr1*, *Dfnb59*, *Slc26a5*, *Lhfpl5*, and *Ptprq*) were changed 2-fold or more (Table 2). Interestingly, expression of those was greater in the apex than in the base. However, there were no significant differences in *WFS1* gene expression.

**Table 2:** Gene expression levels of corresponding genes for non syndromic hearing loss in each cochlea turn.

Gene Symbol	Gene Name [Mus musculus]	Deafness causing Locus	Microarray						Quantitative RT-PCR		
			Signal Intensity Averages			Fold Change			Fold Change		
			apex	middle	base	middle/base	apex/middle	apex/base	apex/base	middle/base	apex/middle
*Pou4f3*	POU domain, class 4, transcription factor 3	DFNA15	200.1	167.3	77.9	2.33	1.09	2.52	3.19	2.59	1.23
*Slc17a8*	solute carrier family 17, member 8	DFNA25	152.3	104.8	47.7	2.36	1.33	3.15	5.07	2.49	2.03
*Tmc1*	transmembrane channel-like gene family 1	DFNA36, DFNB7	90.4	58.5	35.9	1.78	1.42	2.52	2.01	1.97	1.02
*Crym*	crystallin, mu	DFNA40	450.5	301.5	140.1	2.29	1.37	3.14	3.83	2.35	1.63
*Otof*	otoferlin	DFNB9	287.3	210.6	108.8	2.07	1.26	2.61	3.33	2.83	1.18
*Strc*	stereocilin	DFNB16	134.0	80.8	36.0	2.39	1.52	3.65	N/A	N/A	N/A
*Ush1c*	Usher syndrome 1C homolog (human)	DFNB18	211.3	146.7	102.5	1.54	1.33	2.04	1.48	1.39	1.07
*Pcdh15*	protocadherin 15	DFNB23	80.0	50.2	28.6	1.88	1.47	2.76	4.32	1.44	3.00
*Grxcr1*	glutaredoxin, cysteine rich 1	DFNB25	50.9	31.4	12.7	2.65	1.48	3.93	N/A	N/A	N/A
*Dfnb59*	deafness, autosomal recessive 59 (human)	DFNB59	216.5	187.2	86.7	2.34	1.07	2.50	N/A	N/A	N/A
*Slc26a5*	solute carrier family 26, member 5	DFNB61	358.4	201.6	47.0	4.70	1.63	7.65	8.41	5.87	1.86
*Lhfpl5*	lipoma HMGIC fusion partner-like 5	DFNB67	1228.7	902.1	402.9	2.43	1.26	3.07	4.90	2.63	1.86
*Ptprq*	protein tyrosine phosphatase, receptor type, Q	DFNB84	125.0	63.2	30.8	2.18	1.83	3.99	N/A	N/A	N/A

### Tonotopic expression of sodium, potassium, and calcium channels

Many sodium, potassium, and calcium channels were differentially expressed between the basal and apical turns. Specifically, expression of most potassium voltage-gated channels (i.e., *Kcna1*, *Kcna2*, *Kcnab2*, *Kcnab3*, *Kcnb2*, *Kcnc1*, *Kcnc3*, *Kcnd2*, *Kcne4*, *Kcnh2*, *Kcnh5*, *Kcnq3*, and *Kcns3*) was greater in the apex. There was also differential expression of voltage-dependent calcium channels (i.e., *Cacna2d3* and *Cacng2* were higher up in the apex while *Cacng4* was higher up in the base basal). Additionally, sodium channels (i.e., *Scn1a*, *Scn4b*, and *Scn8a*) were differentially expressed between the base basal and apex, and expression of those was greater in the apex. These observations suggest important functional roles for some of these channels in the mouse inner ear.

### Tonotopic expression of other genes important for cochlear function

*Emilin-2*, a major component of the cochlear basal membrane (BM), expressed more in the apex (12.58-fold). Additionally, *Tectb*, a glycoprotein that is localized to the tectorial membrane, also expressed more in the apex (23.85-fold).

### Quantitative RT-PCR (qPCR) confirms microarray data

**Figure 3:**
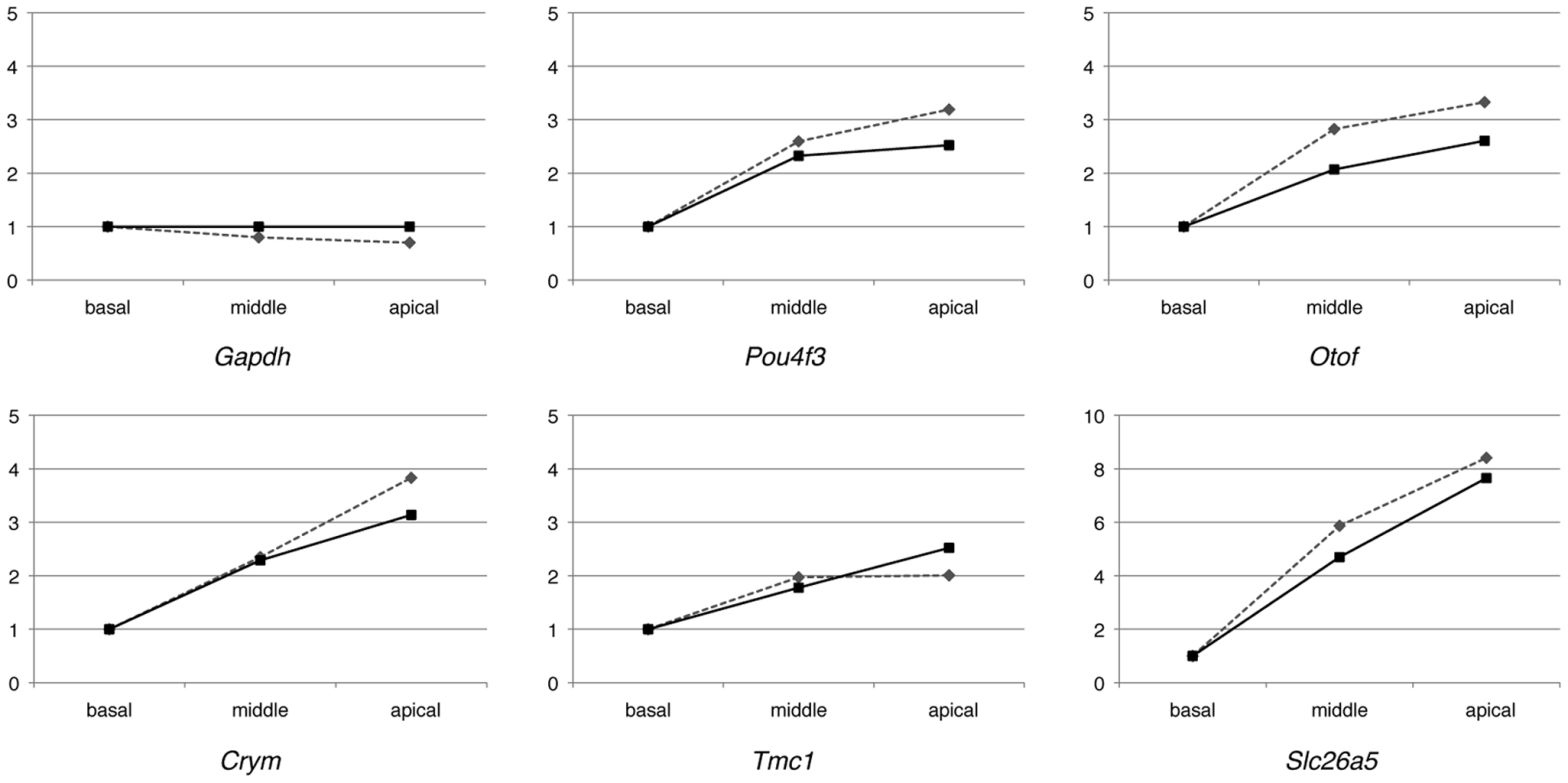
Gene expression patterns found by microarray analysis and quantitative RT-PCR. Values of each gene expression are indicated as a relative value to the basal turn. The expression level of each gene measured by microarray analysis (solid lines) was comparable with the level measured by quantitative RT-PCR (dotted lines).

To validate the microarray data, qPCR primers were designed for of 15 selected genes. Of them, 9 deafness genes expressed more in the apical turn (*Pou4f3*, *Slc17a8*, *Tmc1, Crym*, *Otof*, *Ush1c*, *Pcdh15*, *Slc26a5*, and *Lhfpl5*) and six were internal controls (*Gapdh*, *Actb*, *Rps17*, *Rpl30*, *Atp6*, and *Ipo8*). In all genes, qPCR data was coincident to microarray data. Data of 9 genes together with the control (*Gapdh*) are shown in Figure 3.

## Discussion

These data revealed the baseline of gene expression in each mouse cochlear turn. However, we identified only gene expressions in equal amounts of RNA at each cochlear turn rather than in specific tissue (e.g., the lateral wall, the organ of Corti, and hair cells). This data can be utilized as a tool for global gene analysis such as of the biological function of the genes expressed in the inner ear, or in the search for novel hearing loss causative genes. Sato et al. demonstrated differential gene expression profiles along the axis of the mouse cochlea by cDNA microarray [8]. However, some of our results were not consistent with their findings. This difference may be attributed to the number of microarray probes (165,984 exon probes used in our experiments compared to 20,289 gene probes in theirs). In addition, our microarray analysis results were confirmed by qPCR.

The most remarkable finding was gradients of gene expression, being greater in the apex than the base in ADNSHL genes (*Pou4f3*, *Slc17a8*, *Tmc1*, and *Crym*). There are two prevailing theories explaining autosomal dominant diseases [9]. One of these is haploinsufficiency, referring to a lack of sufficient gene function due to reduced wild-type gene copy number. Cook et al. proposed that haploinsufficiency diseases are caused when the gene expression that is essential to maintain biological function falls below some critical level due to a loss-of function mutation in one of the two homologous gene loci [10]. Many papers supported this theory by quantifying variability in gene expression [9]. If this theory is applied to genes such as *POU4F3*, *SLC17A8*, *TMC1*, and *CRYM*, mutations of these genes would cause reduction of gene products. In such a case, basal turn gene expression may fall below some critical level more rapidly compared with apical turn because of a gradient of gene expression greater in the apex than in the base, resulting in progressive high frequency hearing loss. This speculation is consistent with the reported hearing loss types (such as high frequency progressive) in patients with the *POU4F3* [11], [12], *SLC17A8* [13], *TMC1* [14], [15], and *CRYM* [16] mutations.

*Emilin-2* is a major component of the cochlear BM. The considerably higher level of *Emilin-2* in the cochlea compared to kidney or other tissues suggests a specialized role in the development or biomechanical function of the cochlear BM [17]. Amma et al. considered that if *Emilin-2* confers elasticity on the BM, *Emilin-2* would decrease the rigidity [17] and our results that expression of *Emilin-2* was greater in the apex than in the base may help to explain increased stiffness in BM towards the base.

*Tectb* mRNA expression was 23-fold in the apical turn compared with the middle and basal turns. *Tectb* encodes β-tectorin, a glycoprotein that is localized to the TM and the absence of which leads to disruption of the TM's core structure [18]. Russell et al. reported that *Tectb* ^-/-^ mutant mice, in which exons 1–4 of the gene are deleted, had low frequency hearing loss [19]. Our data that *Tectb* was mainly expressed in the apex, which is sensitive to low frequencies, was consistent with theirs.

In summary, this study demonstrated the gene expression profiles in each mouse cochlear turn. Especially for ADNSHL genes (*Pou4f3*, *Slc17a8*, *Tmc1*, and *Crym*) and other genes important for cochlear function (*Emilin-2* and *Tectb*), gradual expression changes help to explain the findings obtained from previous studies.

## Supporting Information

Table S1 **Gene list showing at least two-fold change in expression in one turn compared to the other turn.**
(XLSX)

Table S2 **Gene list showing at least two-fold change in expression for apex vs. basal.**
(XLSX)

Table S3 **Gene list showing at least two-fold change in expression for apex vs. middle.**
(XLSX)

Table S4 **Gene list showing at least two-fold change in expression for middle vs. basal.**
(XLSX)
